# *Hic sunt leones*. User orientation as a design principle for emerging institutions on social media platforms

**DOI:** 10.1007/s00146-024-01932-0

**Published:** 2024-04-08

**Authors:** Lavinia Marin, Constantin Vică

**Affiliations:** 1 Values, Technology and Innovation, Technical University of Delft, Delft, The Netherlands; 2 Faculty of Philosophy, University of Bucharest, Bucharest, Romania

**Keywords:** Social information, Social media, Social networks, Online institutions, Interface design, Design ethics, Orientation

## Abstract

The phenomenon of missed interactions between online users is a specific issue occurring when users of different language games interact on social media platforms. We use the lens of institutional theory to analyze this phenomenon and argue that current online institutions will necessarily fail to regulate user interactions in a way that creates common meanings because online institutions are not set up to deal with the multiplicity of language games and forms of life co-existing in the online social space. We argue for the need to enable and foster grassroots online institutions that can stabilize the norms of interaction by redesigning algorithms and user interfaces. Such online grassroots institutions would facilitate user orientation at three distinct levels: informational, normative, and semantic-pragmatic. We propose user orientation as a principle that would facilitate the formation of institutions aiming to regulate information exchanges between users inhabiting various forms of life. This principle of user orientation should guide design decisions, while designer teams would need to become aware of the institutional power unleashed when they set up interfaces and algorithms for user-generated content.

## The problem of missed online interactions

In January 2021, Elon Musk tweeted a single word, “Gamestonk!” with a link to r/wallstreetbets,^1^ a subreddit where users discussed non-professional stock trading. After this tweet, the subreddit gained more traction, leading to an increase in the price of GameStop stocks because of the sheer increase in the users who started buying stocks in GameStop. While the artificial price increase made r/wallstreetbets a popular conversation starter and two years later the plot of a movie, we find r/wallstreetbets as an interesting example of a community with its obscure language game, discernible through the memes and slang used by its members. When Elon Musk opened up this community to the general public’s attention by tweeting about it, their language game became visible to a much wider audience. The community’s peculiar language game consisted of a certain usage of memes about “diamond hands”, “hodl”, “stonk”, and pictures of a flying rocket or a nuclear reactor exploding, all related to buying or holding off from selling stocks. These memes meant something obvious to the closed community’s members yet obscure to the outsiders. The memes’ language game meanings were obscure initially, yet the subreddit kept the language game confined to its community where users understood the usage rules, so this obscure language game did no harm. To join the subreddit r/wallstreetbets entailed, by default, learning its peculiar language game. However, once the outsiders learned about this particular meme-based language game, the language game spilt outside and was co-opted by online users in their daily conversations, unrelated to stock trading. This created confusion and strange interactions for people who had never seen those expressions or memes before. A language game carried outside the community where it was formed was bound to create confusion and misunderstandings. Such misunderstandings of user-generated content happen more often than not on mainstream Social Media platforms (SMPs). There is a fundamental difference at stake between these mainstream social media platforms (such as Instagram, Twitter, YouTube, and Facebook) and those platforms where users stay bound within a specific community, such as Reddit. On mainstream platforms, users from various communities meet without context, follow what is viral, and then find themselves misunderstood because of the language games they use or are exposed to. By contrast, on Reddit, each subreddit (community) has its own set of language games, which are often explained in the subreddit rules, pinned at the top, and enforced by moderators and community members.

Language game is a concept put forth by Wittgenstein in order to counter the common intuition that word meanings are universally comprehensible. Instead, the meanings of words and expressions are localized and embedded within forms of life (Boncompagni 2023, p. 6). In order to understand the meaning of a particular language game, we need to look at the community practices and what that language game manages to achieve in that community. A language game, i.e., a particular way in which people use some words to achieve things, gives us an indication of what the community finds useful or valuable: “The usefulness, i.e., the use, gives the proposition its special sense [*seinen besondern Sinn*], the language-game gives it.” (Wittgenstein, Manuscript 131, p. 70, cited from Boncompagni 2016, p. 49) In other words, language games are always “embedded” (Boncompagni 2023, p. 6) in a way of life and reflect what we find meaningful to do. Language does not reflect the world in a word-to-object relation, as was previously proposed in the philosophy of language pre-Wittgenstein; rather, it expresses values and achieves actions. In performing a certain language game, the end goal is performing an action: “the end is not certain ‘propositions’ striking us immediately as true, i.e., it is not a kind of seeing on our part; it is our acting, which lies at the bottom of the language-game.” (Wittgenstein 1977, p. 204) As an example of a language game, when users of r/wallstreet use the meme “diamond hands”, they signal to others that they are holding onto their stocks and not selling despite the price increase. If the meme “diamond hands” is used outside the Reddit community in a private conversation by people who are unaware of the Reddit initial game, the meme can help achieve other ends by changing its meaning and can be an altogether different language game.

Language game-based misunderstandings will also happen IRL (“in real life”): If a stranger on the street wants to hug us, we avoid them skillfully and leave the interaction puzzled. However, what is at stake with online interactions are not mere misunderstandings but incomplete interactions. To explain this difference, we draw from enactive cognition approaches. In an enactive framework, whenever we intend to create a meaning by speaking or acting, we are engaged in meaning-making (de Jaegher 2009). For the meaning to be rendered complete, the others must respond by acknowledging our meaning and thus completing the communicative act through a response. If a stranger on the street wants to hug us, our reaction of rejection and walking away signals to them that the action was inappropriate and thus completes their act in a way that discloses how the action is perceived. In reacting like this, we enable them to understand what is considered by others appropriate behavior so that they can correct or learn it for the next interaction. We are both part of a situation of meaning-making in which we learn from each other shared meanings and the norms of the social world. This simple feedback mechanism of action–reaction–correction creates meaning, even if the initiator of the act did not intend the consequences.^2^

However, in online interactions with other users, the feedback cycle is hardly ever closed. Thus, users often fail to arrive at a shared meaning-making. When the actors involved do not achieve a shared meaning, we designate these as incomplete interactions. When the British singer Adele posted a picture of her weight loss progress on Instagram, she got backlash in the comments but also praise at the same time. Did she infringe on a social norm by posting that picture? She can interpret those comments as she wishes, picking the favorable ones and ignoring the furious ones. The feedback is never consistent since various users are involved in giving it, coming from various social realms and cultures. Meanwhile, the commentators who post angry remarks will fail to receive consistent feedback; they will not find out if their comments were disproportionate, and without this feedback, they will continue believing that it is OK to “call out” someone online and showcase their moral indignation. We need social feedback in a consistent manner to learn social norms and their change. Yet online, on SMPs, the feedback needed for meaning-making is incomplete by design—as we will explain further in Sect. 4, due to the design of algorithms and user interfaces. Human interactions and their success in meaning-making are tied to cultural and social practices that agents share. If one of the actors in an interaction is unaware of the rules, then strangeness and misunderstandings are to be expected.

Given that the rules of social interactions are shared in day-to-day embodied interactions, what makes these rules less salient online? Besides the observation that in “real life”, people do not interact with one another with the same brusqueness as in the online environment, there is something specific about these online interactions in a qualitative way. We see the rudeness as an after-effect of the missed interaction that failed to make a shared meaning and not as the starting point of the interaction. Rudeness as such is merely the effect of users talking past each other and failing to connect in their interpretations of the situation at hand. Many users’ comments are harsh, accusing or demanding, and they do not waste time with niceties or introductions. This rudeness could be attributed to an ethical failure due to the online environment being dominated by a “moral fog” (Cocking and Van den Hoven 2018) whereby users fail to perceive the others as moral agents. However, while illuminating, ethically focused analyses do not fully explain the phenomenon of incomplete interactions that makes the texture of everyday online interactions. Not all missed interactions infringe on common morality, even when brusque or rude. We need a different normative lens to capture the varieties of missed interactions and what is at stake in them for the online social realm.

In this paper, we analyze the phenomenon of missed online interactions and the ensuing misunderstanding through the lens of institutional theory, namely we theorize that the existing online institutions will fail to regulate user interactions in a way that creates common meanings needed for successful communication within the boundaries of a language game. We argue that current online institutions are insufficient to capture the user-generated content that emerges in everyday situations because online institutions are not set up to deal with the multiplicity of language games and forms of life co-existing in the online social space. We then propose user orientation as a meta-principle that should guide the formation of institutions that aim to regulate information exchanges between users inhabiting various forms of life. Our approach of connecting institutional theory with the constraints of language games and shared forms of life is novel insofar as it has not been applied to the online realms of interactions.

## Language games, forms of life, and institutions

From friendship to money, teaching to marriage, institutions are stable conventions put in place by constraints to enable human coordination through automatic behavioral scripts that save members’ cognitive energy (Douglas 1986). Coordination presupposes interdependence and a shared meaning, intersubjective intelligibility and recognition of the same facts and practices. Institutions are bundles of norms or rules, some explicit, others implicit, which implies a distinction between formal and informal conventions. As anyone can witness in their social life, institutions can be imposed from the top-down or emerge from the bottom-up, with some hybrid forms between the two. In the realm of normative dynamics, institutions serve as the codified expressions of shared values, encapsulating the binomial relationship between prescribed norms and enacted practices. The core of this interaction lies in the careful balance between creating clear rules and observing their implementation in daily activities. The capacity of an institution to align agreed-upon norms with the lived experiences of those within its sphere is intricately tied to its legitimacy and effectiveness. Religious institutions typically have established doctrines, scriptures, and moral guidelines. In practice, followers engage in communal worship, rituals, and adhere to ethical principles based on the religious teachings. Similarly, schools and universities have academic standards, codes of conduct, and policies. In practice, students follow curriculum guidelines, participate in classes and exams, and adhere to the educational ethos of the institution. Economic institutions, such as the property rights system, markets, and contracts, shape exchange, frame economic behavior, and set the boundaries of transactions. Cultural institutions, such as family structures, rituals, ceremonies, and gender roles, shape the socialization of individuals, foster a sense of belonging, and define the nature of social relationships. Regardless of these categorical divisions, human agency is enabled or constrained by institutions (Miller 2019); it results from a specific framing created by norms that can be either imposed top-down or emergent from the grassroots.

Linking institutions to a Wittgensteinian framework, institutional norms emerge from shared forms of life (Bloor 2002). We learn to follow rules by seeing and imitating others rather than executing explicit instructions: “to follow a rule is a practice, taught by example rather than by precept within a community of users” (Daston 2022, p. 10). It would seem then that, in order to follow a rule and learn a practice, we only need access to a community of practice. This means that forms of life are prior to institutions: first a form of life emerges and then (not always) the institution that stabilizes its norms and makes its rules explicit.

Feedback from others concerning the norms applicable in each context keeps us on the floating line of social life, saving us from becoming outcasts. Before law enforcement needs to intervene to stop transgressions in interactions, we have already internalized standard norms of behavior in our society through the social response that we receive to certain behaviors. There is an intertwining between forms of life, spontaneously emerging from humans immersed in various communities of practice and the institutions that come later to stabilize these forms. Institutions appear when humans decide that a practice needs stable norms; thus, the institutions fossilize at least partially the form of life and make them more predictable.

Human interactions are fundamentally normative: other community members will judge and decide whether an interaction is failed or successful, appropriate or inappropriate. There are multiple networks of practices in which we are embedded. These practices dictate what is reasonable or not to do and how to interact with others, rather than a global standard. We know in which network of rules we are embedded based on the social feedback from other agents and the social information we can pick up in a particular context. Such networks of practices can be tagged with the Wittgensteinian concept of “forms of life”. Forms of life are the foundation of linguistic meaning, the explanatory mechanism of why we can understand each other because each form of life consists in and is expressed by a variety of language games: we participate in each other’s language games because we share common ways of living, i.e., forms of life. While there are forms of life common to the entire human species—such as eating or exchanging verbal interactions—there are various localized forms of life that only a community of practice can access: “Whereas all humans share in a fundamental form of life, there exist, within this shared biology, behavior and environment within these shared ways of living and (as we shall see) patterns of life—possibilities for diversity and variation; for, that is, various forms of human life.” (Moyal-Sharrock 2015, pp. 25–27) Thus, while we share the same human form of life with aboriginal hunters in some remote island of the Pacific, we cannot understand their language games about hunting practices (visible in signs, gestures or words) just as they could not understand the memes used on some social media group. The forms of life are too far apart and the language games cease to have meaning for these two communities. Because the forms of life are so radically different between the hunters and the social media users, we can easily understand why the language games are incomprehensible to each other. However, the situation becomes more complicated when various groups of social media users interact on the same platform. Do they share the same “social media form of life” or can we discern here various forms of (online) life with their special language games?

Formal or informal institutions—comprising regulative, normative, and cultural–cognitive elements—structure and enforce behavior by offering stability and meaning to social life (Scott 2014, p. 56). However, as we will argue next, social media platforms have a problem with institutional power because the existing social institutions fail to stabilize the emerging norms, due to the multiplicity of such norms emerging from various forms of online life. There is a variety of forms of life found on social media given that SMPs are spaces where people from all over the world can meet. Consequently, the norms that users think they should follow when interacting online are also multiple and unpredictable. Hence, it would seem that the main problem with online interactions is that it is unclear which forms of life participate in an interaction since online communities do not have clear boundaries (Marin, 2021). The only visible demarcation between forms of life is language, but when various language games occur within the same language community, it is difficult to detect this clash in a reliable way.

When two language games clash in an interaction, one could point to the incomplete interactions and misunderstandings among users as a surface symptom; a more profound issue is the disorientation of users between what norms and practices they should follow and when. An online community may decide today to use a word such as ‘woke’ to signify something positive or negative, carving its local meaning, and will do it consistently if most of its members agree. However, on SMPs, that community has no way of delimiting itself from other communities since its members cannot signal explicitly when they play the language game, and singling out who is a member and who is an outsider based on language games alone becomes difficult (one can think of irony or sarcasm). This is how dog whistles and emojis used as signifiers for allegiances work online (Alfano et al. 2021): a new language game without boundaries that spills into the shared pool of language games creates confusion and misrecognition for other users unaware of the convention. What looks like a language game problem is, we argue next, an institutional problem. The existing online institutions are too weak to stabilize language norms, so the language games map to the social information^3^ continuously generated by users.

If existing institutions found online are too weak, it seems that we need new online institutions. How should these new online institutions emerge? In the next section, we argue that there is a minimal guiding principle that should oversee the emergence and formation of such online institutions. This principle is normative, but it should not serve a specific value or form of life since this would hinder the diversity of values embedded in forms of life out there, thus alienating users from other cultures or communities. Instead, this would be a meta-principle for the design of online institutions that will not interfere with the forms of life being stabilized by not adding its own values and implicit norms to the content of what is shared among users. This is what we term the principle of *user orientation*.

## User orientation as a meta-principle for design

To be able to deal with the normative complexity found through the variety of forms of life that they are exposed to, users of social networking platforms need both to make sense of long-lasting practices that stabilize as institutions and to create a hierarchy among those practices by selecting which norms should be followed in the context of each online interaction. Currently, neither option is readily available, hence the widespread disorientation and misunderstandings. Users need to have a way of orienting themselves among the competing norms such that users understand which norm is achieving which social good in a particular context. We put forth the principle of user orientation as the overarching principle that should guide any design choice when making online spaces where users produce and exchange social information.

Our take on orientation as a guiding principle is different from, for example, value-sensitive design approaches (Friedman 1996) in a fundamental way: we do not aim for orientation as a value to be pursued universally; instead, orientation is a constitutive principle for how social spaces should be designed. Orientation should allow users to coordinate with others from the same community to pursue particular values and detect when they are playing different language games and thus are encountering a new form of life in their interactions. While value-sensitive design starts by zeroing in on the community of users for which the design is made and inquiring about their values regarding a specific technology, our principle of orientation aims to account for the fact that online, users do not stay put in a community and will face other communities constantly, hence that the values cannot be in principle designed for nor anticipated before the interaction takes place. Assuming this structural impossibility to design for the encounter of forms of life, we aim for the second best option: an awareness that there is such an encounter.

Orientation is about finding one’s bearing. Our choice of orientation as the main principle is not accidental; we think it has the potential to elucidate precisely what online users are missing when trying to function in the online lifeworld. In one of his lesser-known writings, Kant advanced the concept of orientation, extending it from geography and mathematics to orienting “in thinking in general”, i.e., logically (Kant 1998, p. 5). Kant aimed to elucidate how pure reason can guide itself when it leaves “familiar objects (of experience) behind, extending itself beyond all the bounds of experience” (Kant 1998, p. 5), so beyond any object of intuition, toward the supersensible. Inspired by Kant and extending the Kantian framework, we advance another conception. For Kant, the challenge was logical orientation; for us, the concern is with *informational* and *semantic–pragmatic orientation* (in the case of language games). In the case of online user interactions, design mediates between sensibility (perception) and understanding (intellect). The role of designers is to frame the users’ perception to help the intellect make a suitable concept–object identification. In other words, to make digital objects as familiar as possible to the human experience by helping users discern the social information surrounding digital objects and the norms of interpretation of said information.

Orientation works hand in hand with the practice of navigation. The metaphor of navigation has already been used earlier to describe what Internet users were doing (Hochmair and Luttich 2006). For Dreyfus, ‘playful surfing’ was the specific mark of the digital culture, as opposed to ‘interested browsing’, which was the activity of the library culture—showing that people not only collect but also connect online information (Dreyfus 2001, p. 11). However, as Web 2.0 gained ground, this metaphor has been abandoned for the competing metaphors of users as consumers and, at the same time, users as creators. We think that navigation needs to be taken up again as defining what users do in a move away from the image of users as passive consumers. However, not all navigation modes are equal, and the possibility for orientation needs to be designed within any navigation for online users and their interactions. Navigation as a metaphor opens up a new understanding of what online users can do: navigation is a complex task, with multiple points of failure and possibilities for backup. Navigation is an exercise of positive and negative liberty, constitutive of agency (setting and pursuing goals). Navigation gives the users moral agency, but this moral agency must be constructed through conditions of possibility, which are given by what users can do with social information found online. Online orientation in navigation is orientation in massively social information.

We distinguish between three ways of understanding user orientation, two of which have been theorized in the previous scholarship: visual orientation within the information available (as proposed by Christian Vandendorpe), normative orientation as ranking used for evaluating the information and assertions found online (inspired by the work of Gloria Origgi); and, to these we add a new kind: the semantic-pragmatic orientation, which aims to help uses stabilize the boundaries of language games enacted online, and thus find meaning. We explain briefly the three kinds of orientation for users below.

### Visual orientation

According to media scholar Christian Vandendorpe, two significant ways of visual orientation compete for the user’s attention online: the non-linear, map-like way of the codex and the linear scroll-like way of the papyrus (Vandendorpe 2009). While the first way demands that the users take an active role in navigating by choosing a purpose for one’s navigation and following what interests them, like explorers following a pre-established goal, the latter way puts the users in a primarily passive mode of interaction: users will simply scroll down and encounter unexpected information deemed relevant by the algorithms of personalization. Thus, Web 2.0 users no longer need to search for relevant information; they receive it right where they are and only need to scroll down. Navigation is replaced by a passive reception mode in which the information is served to one’s eyes right on time.

Every media revolution is characterized by a new kind of orientation within information. What kind of visual orientation was made possible with the World Wide Web? The World Wide Web emerged with two distinct information architectures: the hypertext and the scrollable page. The hypertext took non-linear reading to the next level, allowing users to jump between pages or sections while clicking links. The medieval codex was the direct inspiration for the hypertext, embedding its values of accessibility and orientation, bringing map-like exploration of information to a new level. However, while the hypertext is tabular, the actual web pages are experienced as unfolding scrolls. We read web pages by scrolling down, following the text where it leads us unless we choose to click on links and go elsewhere. This should not be a problem by itself; scrolling down is necessary when the text does not fit into a page so we can navigate visually by skipping bits. However, with the advent of social platforms online, where the users are the main content generators, the linearity of the scrolling down came back with a vengeance. With social media platforms, we are back to the linear access to information of the papyrus, but this time it is an infinite scroll. The linearity of the navigation on these platforms was a deliberate design choice to enhance features such as personalized content and showing adverts in a more visible way. This design choice was neither good nor bad, but still, it is a choice that structures one’s cognitive experiences with online information, and we need to be at least aware of this choice.

### Evaluative orientation: as ranking

When we evaluate other users online, we may use various scales for popularity, epistemic credibility, moral virtue, etc. Most of the evaluations we do as users online stem from these ranks that we find or are constructed for us. For example, a metric intended for measuring online visibility is the number of likes or followers a user gets. It should not be used to evaluate that user’s epistemic trustworthiness, albeit often this is the case. Following Gloria Origgi, the Web—and SMPs as a part of the Web—“is not only a powerful reservoir of all sorts of labeled and unlabeled information. It is also a powerful reputational tool that introduces ranks, rating systems, weights, and biases into the landscape of knowledge” (Origgi 2018, p. 193). The work thus far done in the epistemology of social media has focused predominantly on how these ranks work as proxies for trust and epistemic credibility. Making a broader epistemic point, Origgi argues that we first compare and rank in order for us to know: “to be is to be compared overturns the classical conception of knowledge according to which awareness of an object of knowledge precedes its evaluation (…) we evaluate in order to know, meaning we have to locate the objects of our knowledge in an evaluative system so that we can compare them with each other” (Origgi 2018, p. 243). Ranking online is a form of user orientation, perhaps the most basic one, since the metrics for ranking are so easily accessible and comprehensible to all users across various societies and cultures. This ease of ranking relates to the gamification occurring in most systems that provide quantifiable metrics, such as likes, followers, reposts, etc. The gamification aspect has been discussed extensively in the work of Thi Nguyen (2021), predominantly with Twitter as a case study. Nevertheless, what interests us here is that almost no mainstream SMP is without these quantifying features. Thus, user’s orientation among online influencers—choosing whom to trust, whom to like, and whom to follow for information—usually is based on evaluating and ranking such influencers. Thus, there is an orientation at this basic ranking level, yet we argue that this is insufficient. We also need a way of orienting ourselves among the communities that we cross through our online journeys on SMPs, we need to know where we are, not only who is the most famous voice in this particular community.

### Semantic–pragmatic orientation

A multiplicity of language games and forms of life makes it hard for users to understand what language game they are participating in, the rules for playing it, and when exactly they switch between forms of life. Thus, equally crucial as orienting oneself visually in the massive online information or as evaluating the most trustworthy source, users need to have a clear way of navigating between forms of life and their associated language games and between the institutions to which these forms of life give rise. The matter becomes complicated because we experience institutions and forms of life online primarily as informational transactions. Hence, information orientation is the main way to design for an architecture of plurality, yet the aims of designing the user’s experience should not be merely for the understanding of information (semantic) but an orientation among forms of life and the institutions attached. Users process information online at three levels, which are connected: “not only technological (e.g., affordances) but also individual (e.g., selective exposure) and social (e.g., sharing practices)” (Reviglio and Agosti 2020, p. 2). Based on this terminology, we are primarily interested in how users can be oriented in the massively social information (especially in the practices they share, but also forms of life, language games, and institutions). In addition, we also recognize with Reviglio and Agosti that social information cannot be disconnected from the individual user information and from the technological affordances which make certain kinds of information more visible than others.

We have thus far proposed that the user’s orientation is fundamental for navigating between forms of life found online and that this orientation cannot be only visual and evaluative, two forms of orientation thus far already theorized, and that it also needs to be semantic–pragmatic. The next concern is how to ground this orientation and ensure it is structurally part of the user experience on SMPs. In the next section, we argue that orientation, as a meta-design principle, is made possible through design choices at the level of algorithms and user interfaces. However, these design choices make possible a new class of more robust and more stable online institutions.

## Algorithms and interfaces: two candidates for online institutions

### Kinds of institutions found on social media platforms

We take the primary function of any institution to offer stable constraints for creating order (North 1991) and predictability (North 1990) for the multiplicity of forms of life existing in any community. In short, like information, institutions reduce uncertainty (North 1991, p. 97) by offering classifications based on analogies, that is, a cognitive^4^ (automated) toolkit for exploring the world (Douglas 1986, p. 112). A lack of institutional normativity entails ineffective agency in social life, even if individual agents may be free to act as they will. A world is genuinely social only when institutions structure it; otherwise, it looks like a (more or less) messy set of nominal entities and their unpredictable relationships and roles. Some norms arise immanently in the social world (Rouvroy and Berns 2013) while others are imposed by institutions from top to bottom. Therefore to detect whether there is an institutional void we cannot look for normative voids, since norms are always present in any common social spaces. Rather, we need to look at the forms of life forming around these norms and at the agreements in language games, what we called ineffective agency: when language games fail to turn into actions. If there is misunderstanding as a rule and incomplete interactions, norms are ineffective and we can suspect a wider institutional void behind this phenomenon (see also Vică and Socaciu 2019).

Our proposed model of language games is not the only one aimed at understanding the specific nature of SMP and how ineffective agency occurs due to institutional failures. There are currently many theories competing to address this general issue, such as ‘surveillance capitalism’ (see Zuboff 2015, 2019), which focuses on how social media platforms commodify user data for economic gain; algorithmic governance approaches (see Rouvroy and Berns 2013), which focus on how algorithms influence the visibility of content, shape user experiences, and affect the distribution of information; or the more recent content moderation theory (see Gillespie et al. 2020), which focuses on how platforms enforce rules, handle user-generated content, and strike a balance between freedom of expression and the prevention of harmful content. Our model looks explicitly at what lies behind governance, the real language game, and human practices. That’s why we pay attention to informal institutions (and how difficult they are to design online) in the production of shared meaning, rather than the powerful top-down forces such as algorithms (which we recognize as de facto shapers of interaction—see 4.2) or the economic structure of SMPs as firms in the market.

It has been argued that there are very specific online institutions at work, visible in how profiling and big data algorithms work to personalize information, giving rise to an algorithmic governmentality which “‘creates’ a reality at least as much as it records it.” (Rouvroy and Berns 2013) Even if algorithms create their new norms and some novel institutions,^5^ one can still wonder what happens to the old norms and forms of life that get carried over by users into the online realm? When social norms carried over from the offline world clash with the new algorithmic norms, we face a difficulty in finding common normative grounds for distinct language games. Strong norms are desirable but hard to find when various life forms interface and interact, especially when the online and the offline forms of life compete on the same terrain.

The fact that online social information gets interpreted unpredictably, leading to missed interactions and misunderstandings, does not mean that institutions are absent from social media platforms. Instead, these institutions are weak, unstable, thus unpredictable, and cannot sort the language games that users play into various communities of practice. Prominent formal institutions online are the Terms of Service that users agree to (usually without reading) when joining a platform. These terms are institutions with legal force and can be used to exclude users from specific platforms or to hold them legally accountable for hate speech or other illegal acts. These terms of service concern what a user *should not do*, hence acting out as boundaries for behavior, as top-down institutions. Nevertheless, what they do is left to their interpretation of norms and practices.

When multiple forms of life emerge bottom-up, the ensuing normative complexity becomes problematic only if no institutions are in place to stabilize and separate these forms of life. ‘Classical’ institutions (like rituals, customs, or laws) managed to direct, orient, order, and give (mental and physical) space for human agency. This is now made possible by digital platforms; however, rather than public understandings of normativity emerging from recurrent social practices or political authority, privately owned platforms control and orient human motivations and intentions, rhythms, customs, and collective habits and routines of life (Vică 2023, p. 154). This situation entails a risk: when technology-based institutions replace some social and even political institutions as surrogates, we can expect the growth of dis-coordination and the loss of shared meaning (Vică 2023, pp. 155–156).

Given the normative complexity and the vast amounts of social information that users face, it becomes clear that users need not only enforcing top-down institutions that restrict their actions but also institutions that organize the social information that users create, namely grassroots institutions. Such institutions would need to constrain user behaviors and interactions, but not by placing interdictions and blanket rules. What we call grassroots institutions are those that emerge and that develop naturally without being imposed by an authority. Such institutions are usually found in social life. Family norms, friendship rules, neighborhood helping, giving priority to expectant mothers—are all mechanisms for coordinating and guiding behavior that ensure social stability. Rules and norms are often implicit or informal, learned by observing what others do and through repeated interactions.

We also see some emerging informal institutions online that have the capacity to constrain user behaviors and interactions but have not yet been fully explored to their potential. In the next section, we explore algorithms and interfaces as the new emerging institutions for the online world. Even if algorithms and interfaces do not emerge “naturally” at the grassroots level from repeated user interactions (although they evolve and change precisely because of them), even if they are imposed by a private authority that owns the platform, their function is, in fact, equivalent to that of implicit, informal social institutions. As we will see below, even if they have an obvious regulatory function, they are placed in a zone of informality, making it difficult for users to understand how they function and act on the possibilities of online expression.

There are some powerful structures of SMPs that can take on the role of stabilizing institutions. In the next section, we discuss two of them, algorithms and user interfaces, while acknowledging that these are not the only two options, just the most obvious ones.

### Algorithms as institutions

Algorithms de facto shape how interactions occur on SMPs. Not only interaction with the communication and design elements of the platform but also with other users participating in various language games. Algorithms generate the totality of interaction possibilities (which is, granted, a limited set) from often vast and unnavigable content. This is why they are often recognized as artifacts with agency, even autonomous actors (Just and Latzer 2017, p. 253) that can shape users’ interactions and are shaped by them. However, this perspective shifts the focus from their role in *regulating* user activity, which is their primary role. It is not *what* algorithms do that is important, but *to whom* they do it. Moreover, more importantly, *how* they do it. Through a series of steps, algorithms function like any Institution by embedding rules that channel certain practices in action and make it impossible (or very difficult) for any behavior to deviate from the program. They are stable in one sense—being unavoidable and sorting out possibilities—and unstable in another sense—their output can vary depending on the previous choices and actions of the users and their connections. However, just unlike institutions, they do it without the user’s knowledge and awareness—the average user does not know why they can interact with something, and they do not know what they cannot interact with. Imagine a state where the citizen does not know how the rules work but is still forced to follow them and cannot “escape” their dictatorship. Apart from the fact that this has not happened as such in totalitarian states (where citizens know how the rules work and why they should not be broken because they would lose all freedom), such an image shows the algorithm’s total blind power over users. Moreover, users of the platforms are subject to constant surveillance, which generates a wealth of data and makes algorithmic action possible. This would be one reason why even the category of ‘totalitarianism’ does not capture algorithms’ true power.

Algorithms are still the primary candidate for online institutions that can equitably govern users’ interaction on SMPs. They de facto exercise the “governing power” (Lazar 2023, 11:20): “Our experiences are governed by algorithms that are constantly monitoring and shaping our behavior and our attention, automatically selecting what we do and do not see” (Zimmermann et al. 2022, p. 1). In this case, the problem becomes acute: their power is recognized, but their normative effects on users’ lives are not controlled. They perform the function of institutions, but their mechanisms are hidden from us, and their investigation is almost impossible, both because of their unstable nature and the secrecy or opacity that protects them. Hence the need to make them transparent, (self-)explainable or auditable, etc. This we identify as the actual challenge for the whole digital realm, not only for SMPs but for any other environments with user-generated content, from search engines to online crowdsourced encyclopedias to collaborative video games. And even far above, in the whole social life.

The asymmetry of knowledge between algorithms and the subjects of their governance does not necessarily translate into technological determinism, which would also be impossible due to their unstable nature but is one reason why their power is not institutionalized for the real benefit of users. The co-evolution of algorithms and behavior is not the result of intentional and explicit co-creation but of a de facto domination of the platform over the users’ ability to orient themselves in (cyber)space and in the world, reducing the chances of a shared meaning. A redesign of algorithms, especially those resulting from machine learning, to restore a horizontal plane of co-creation is not possible if users are not directly and knowingly involved. If agency is distributed between users and algorithms, normativity should be equally distributed. This is where SMP owners and designers can intervene. They can make it transparent and explain to each user how the algorithmic action takes place even in a comprehensive visual way. Thanks to some legal institutions, platforms must explain to users what happens to the data stored by the platform. Of course, this explanation is sometimes unlikely, either because of machine learning limitations or users’ epistemic limitations. But like any language game, it can emerge, be learned and maintained. Its practice, the continuous process of understanding what is happening, has a high chance of stabilizing communication and cooperation on platforms. It will certainly respect the principle of autonomy and human dignity.

### User interfaces

User interfaces are sets of designed affordances, such as the buttons on which the user clicks, the fields one can fill in content, and the graphical layout that arranges the information on a web page. Affordances are possibilities for action organisms perceive in their environment (Gibson 1979). A chair is perceived as an affordance for sitting, a knob is for turning, a button is for pushing. Based on Donald Norman’s concept of designed affordances (Norman 1999), digital affordances have been classified into three kinds: perceptible, hidden and false affordances (Gaver 1991). All three kinds of affordances are interacted with via user interfaces.

For social media users, perceptible affordances are those they can directly perceive, such as buttons and links that one clicks on, text boxes that allow writing, and placeholders to upload images. The hidden affordances are for the tech-savvy, and these concern fiddling with the settings menus to display the content differently or installing third-party apps to modify the page’s source code. Hidden affordances also create a personalized environment through algorithmic decisions based on the user’s actions: choosing to click on certain stories will lead the algorithm to feed the user with more of the same type of stories. These are hidden affordances because the user does not have clear control of them (as was the case with perceived affordances) and can only infer those affordances. False affordances are those that mislead the user into believing this is an affordance, such as clicking on a button that does nothing or filling in a form that seems legit but is actually a scam for collecting personal data for other purposes. False affordances are outright errors of coding or immoral moves. However, in our quest for providing beneficial user orientation, we advocate for more perceptible affordances and for making the hidden affordances more visible and accessible. Nevertheless, affordances only work at the level of content. We need more than easy navigation within the informational content to arrive at orientation.

Just as a speed bump can slow cars on the street and enforce the speed limit, online interfaces can enforce certain rules of behavior for online users. Here are some examples: one can imagine an interface for posting comments that do not allow users to immediately post a comment, asking them to wait and rethink it, maybe for 15 min. Some Instagram algorithms perform a sentiment analysis on the comment and ask the user if they are sure they want to post a comment that sounds hateful or discriminatory. A Twitter design intervention during the 2020 U.S. presidential elections asked users to retweet with comments, thus making the use of the retweet function less frictionless.^6^ This design feature was meant to slow down the rapid spread of information and make users explain their reasons for retweeting something since the tweet itself does not explain much. These kinds of interface tweaks—delayed comments, sentiment analysis of comments and posts, or retweeting with a comment—allow the user time to reflect and consider what they want to say and ultimately remind them that they are in a public space after all. Just as a user interface can slow down or accelerate reactions, it can foster certain modes of cognition (affective or reflective, intuitive or conative), and it can also enforce certain norms, albeit the question remains what those norms should be.

Interfaces can act as enforcers of norms by tweaking the affordances set in place or by hiding affordances from users. However, this enforcement role is not always acknowledged explicitly by the designers of interfaces. The norms and practices promoted by interface design are left to a common understanding of appropriate user interaction, which usually maps over a Western understanding of usability and functionality. These norms need to be up for debate in the design teams but also need to be acknowledged by users explicitly. Our argument is not that interfaces should not be enhanced or redesigned in response to specific problems identified online. Instead, suppose we acknowledge that interfaces and algorithms can act as institutions, enforcing or discouraging online practices and norms. In that case, we need to provide online users the ability to see that and choose the forms of life they want to participate in online. In other words, this versatility of online forms of life emerging in the same spaces and the online quasi-institutions, designed based on principles that are not discussed, gives rise to a specific problem of online spaces that other social spaces, which are not that heavily designed and planned, do not seem to have. The problem is one of disorientation caused by the inherent normative complexity: users do not know what rules and norms apply to the situation at hand because they do not know how the unseen audience of online others will interpret their actions. The social and ethical space in which we interact online is not clearly demarcated by any language game or community of practice. This is a kind of normative disorientation: not that we do not know what is right or wrong, but rather the context in which our actions will be judged becomes unclear as its boundaries are fluid. Marwick and Boyd (2011) introduced the concept of “context collapse" to explain how, on Twitter, the context of a tweet is not carried over when that tweet is retweeted further, making it easy to lose its meaning. Similar to the context collapse on Twitter and extensively on other social platforms, we also notice a context multiplication: online audiences bring their own contexts of interpretation and multiply the interpretations of a message unpredictably.

## User responsibility and techno-deontic powers of design teams

Thus far, we argued that the missed interactions between users on SMPs can be traced back to the amount of massively social information shared by users who enact language games opaque to other users. A solution we proposed to this “Babel tower” of language games was to look into the potential of institutions to stabilize emergent forms of life. However, as we tried to show, SMPs lack bottom-up institutions and only have top-down institutions in place. Then we asked: What structural features of SMPs would allow them to act as grassroots stabilizing institutions? Given their power to shape user interactions, we zeroed in on algorithms and user interfaces as two plausible candidates. Should we then redesign algorithms and interfaces for more institutional power and accountability? Our answer is affirmative, but this design needs to be deliberate, considering the institutional power unleashed when interfaces and algorithms are designed.

Our solution to online users’ disorientation between forms of life entails establishing new online institutions by redesigning algorithms and user interfaces to facilitate user orientation at all three levels: informational, normative and semantic-pragmatic. While we know how to facilitate visual orientation or normative orientation—by adding, for example, page breaks or ranking trusted users in a domain “verified user”—the semantic-pragmatic orientation is still difficult to envision. We put it out there as a meta-design requirement that design teams should consider and plan for whenever they redesign mainstream SMPs. While we do not know yet what designing for semantic–pragmatic orientation could look like, other than perhaps starting by opening up the design decisions to wider communities and publics, we envision at least three approaches to design that could lead to enhanced user orientation. The first approach is to design worlds as open as possible, accommodating a multiplicity of forms of life while, at the same time, helping enforce norms via stable and predictable structures of interaction. Second, one should aim to facilitate understanding and cooperation by making social signifiers as visible and explicit as possible, even guiding for ideal situations, such as online conviviality (Voinea 2018). Lastly, designers should aim to enhance the user’s agency by designing affordances for orientation within the social information content and various forms of life that will give the norms for interpreting said social information. This presupposes designing for user autonomy and self-reflection by reminding users of their values and professed self-identity while leaving clear options for possible self-development.

Using Searle’s concept of deontic powers (Searle 1995), which are “rights, responsibilities, obligations, duties, privileges, entitlements, penalties, authorizations, permissions” (p. 2) and whose purpose is to “regulate relations between people” (p. 100), we propose a similar concept for the online lifeworld that we call ‘techno-deontic powers’. Techno-deontic powers over a digital platform produce rights, obligations, entitlements, privileges, penalties, permits, bans, etc., just like the ‘classical’ deontic powers, or functions, in Searle’s vocabulary (1995, p. 100). Designers have what we call “techno-deontic powers” over the users of the platforms precisely because they acquire a scarce kind of knowledge, “algorithmic knowledge” (Solcan 2003, p. 71), that makes them able to invent or discover, then put to work and supervise algorithms. Unlike the classical deontic powers, whose sources are institutional facts, the techno-deontic powers we envisage have a different origin. This kind of power is conferred neither by a collective recognition of their status (a mechanism described by Searle 1995) nor by political authority (based on a social contract) nor by the collective intentions expressed by users. The source of techno-deontic powers derives from a closed, often proprietary knowledge of the platform’s algorithms and interface design. Digital world-making is based on algorithmic knowledge used for human institutionalization, norming behavior and setting “normal” or standard boundaries in intelligibility and cooperation in a top-down manner.

The concept of techno-deontic power entails that the responsibility for what happens on social platforms is larger for designers than for users because the latter will always experience a limited agency precisely because of design constraints. This entails that particular design duties stem from the fact that a creator of worlds is also an experience enabler since the artificial worlds offer the conditions of possibility for some experiences but not others. Designing experiences, a mantra in the web industry, should be understood primarily as a morally laden activity of creating institutions, and it should never be reduced to a pursuit of technological novelty and efficiency. In Kantian terms, as world makers in our post-digital era, the designers are in the business of transcendental esthetics: they should understand the delicate relationship between sensibility and understanding (intellect), between perception and judgment, and between intuition and concept. Design choices for both interfaces and algorithms could, voluntary or not, disconnect these two faculties of knowledge or make them work together perfectly. The design could steer sensibility to lead to mindless, hateful or manipulative behaviors, as seen with some Facebook or Twitter (X) incidents, or it could integrate user’s sensibility into the higher demand of understanding and categorical thinking like Wikipedia attempts to do. This decision boils down to what we have called a duty to *design for orientation*. This does not mean however that designer teams should prioritize a priori a form of life over another, deciding from their own cultural background what should matter for the users. Rather, we want to leave the decision to the users themselves, while keeping the designer’s intervention limited to making users aware of the multiplicity of language games in which they immerse themselves each time they go online. The gist of our proposal is to increase the SMP user’s agency by making them aware of the multiplicity of language games, while taking away from the designers any duty to prioritize among language games.

One objection that could be raised to our proposal concerns our focus predominantly on the power of designers to create such orientation, given that design is made in teams (usually massive teams) complying with decisions from the managerial side, often in huge corporations. What is the actual power that designers have to influence the design of SMPs? It would seem that very little power in actuality. In this paper, we are not concerned with who makes the actual decisions in the design process since this is a socio-technical issue with multiple actors pitching in, negotiating and deliberating; rather, we are interested in highlighting that the actual practice of design has the power to shape worlds, regardless of how is taking those decisions. Even if the design decisions come through a long chain of managerial decisions, with complex negotiations and back and forth, even if such decision may be said to “emerge” in the negotiations, we are still concerned with the power of design to shape the social world, regardless who is behind that decision. Even if the designer decisions become at some point collective and corporate, the power behind such decisions needs to be highlighted as clear responsibilities should follow for those in charge of design: companies, managers, teams, or individual designers.

It may seem out of sync with contemporary realities to offer grounds for a moral conviction such as ours: designers have a duty to guide users, or at least a duty not to disorientate them at the crossroads of different language games. In general, designers are not owners of SMPs, and they cannot freely choose which (moral) principles of design to follow. But this does not absolve them of any responsibility: their techno-deontic power is a double-edged sword. They shape users’ worlds, institutionalize their practices (or fail to do so), etc., and still cannot cut through corporate or even political control. However, even when done by teams, design is a world-making choice because it is about setting up user experiences. Tim Berners-Lee once called the information architects behind the web technology “philosophical engineers” (Halpin and Monnin 2014). The philosophical engineer is the one who deals with the design issues of a specific information system (Halpin and Monnin 2014). They interpret, forecast, and modify the technology. Users also play a part in how the online worlds are structured, but their capacities are framed and constrained by design. Treating user experiences in a *laissez-faire* manner has led to high cognitive burdens without cognitive enhancement effects (Voinea et al. 2020). It also instrumentalizes users’ behavior by steering them away from their self-directed goals and toward spending their time and attention to benefit the platform (Voinea et al. 2024). This behavioral hijacking leads to a gradual autonomy loss (Voinea et al. 2020) and thus to a weakening of their social performance.

The question most salient for designers—how to put affordances in place, such as to foster a more oriented behavior for users—cannot be answered fully a priori. Rather, we must look at how communities use digital affordances, how they socially signal to each other the possibilities for action and then make these social signifiers as visible as possible. This also means the designer’s task is not finished when a design is completed. Rather, the designer must return and adjust the system according to how the system is effectively used, which also means interfering with algorithms, which are anything but objective or neutral. This fundamental indeterminacy on the user’s side of the experience introduces new duties for designers: they cannot design for all possible uses and should not try to. Rather, designers should make possible the user’s navigation between different forms of life. It means, concretely, that when users switch between two forms of life found online, they need to be able to grasp instantly that first, they are witnessing conversations belonging to a different form of life, and secondly, what are the institutions at work there (especially the bottom-up norms and tacit rules), what are the language games in use. This is impossible to do without noticing how other users play the language games and how they use the affordances. Hence, social signifiers must be put into place, but also a clear signal for users to know when they are switching between forms of life. The current architectures of social media, based on infinite scroll of various posts, clearly do not allow for that. While scrolling, you encounter different language games with various new posts, but these are all homogeneously shown to the user, making it hard to understand what others mean with their posts. This unintelligibility of others makes it such that emerging online institutions have very weak norms and become hardly effective.

What about the responsibility of SMP users? Is being oriented as a user a surefire solution for behaving more responsibly online? We do not claim that user orientation is a sufficient principle for having a comprehensive ethics of design for online environments, rather that it is a principle thus far neglected since it was not conceptualized. We claim that any design ethics concerning online social spaces needs to consider this principle, particularly when such spaces are constituted by user-generated information. Thus, while user orientation is not enough by itself to ensure ethical interactions, it is necessary for intelligible interaction and a pre-condition for establishing a shared normative space. Users of SMPs are not free to pursue whatever value they choose online as individuals. Instead, the values we pursue are almost always dictated by the community, as there is something fundamentally collective in the nature of values (Van den Poel 2013). To achieve clarity about the values, we may want to fulfill through our online interactions, we first need clarity about the community we belong to, its norms for interaction online, and its social goods pursued in each context, even if it abounds in massive social information. Without this clarity given by orientation, we will fundamentally find ourselves undermining our community with our actions, and our expectations of participating in a shared form of life online will be repeatedly sabotaged by the platforms’ incomprehensible or opaque institutional monopoly.

In medieval times, maps used for navigation had the inscription “Hic sunt leones” for areas of unknown and potential danger. For the uncharted territory of online social spaces, the question is no longer where lies the danger; rather, the users are concerned when they cross an invisible boundary between forms of life and language games. Such a crossing cannot be signaled properly, visually or in some normative sense (e.g., signaling ‘our’ communities from alien ones). Rather, users need a new understanding of what orientation is and, for this, a way of grasping when they are speaking with other members of online communities and of discerning when the language game has changed. We need a new version of the “Hic sunt leones”, not as a warning to stay away but rather as an invitation to enter new social spaces in which people who think and live differently have something to share and from whom we can learn.

## Data Availability

No data is associated with this manuscript.

## Footnotes

1 See https://www.cnbc.com/2021/01/26/gamestop-jumps-as-elon-musk-tweets-out-reddit-board-thats-hyping-stock.html

2 However, not all missed interactions and negative feedback loops are due to misunderstandings in language games; sometimes, it is simply the case that a social norm is tested, and it is up to society to enforce that norm again and again. For example, sometimes, the initiator knows their actions are not wanted, such as pickpocketing tourists on a busy street. In this case, it is not a misunderstanding of meaning-making but perseverance: despite the adverse reaction of their victims, pickpockets will keep trying to reach their goal. Pickpockets know fully well that their actions are seen as socially inappropriate, and their perseverance in these acts is not because of a lack of social feedback but because the social counter-reaction is not effective enough.

3 Social information is information we gather from those around us to learn how to behave (Baldwin and Moses 1996). Social information serves two primary purposes: for individuals, to help them fit in their community by learning the accepted ways of behavior, and for the community, it helps with pursuing common goods such as coordination, solidarity, safety, etc. For social information to be used toward common aims, the participants need to be under the impression that “this information [i]s representing the behavior of a ‘generalised other’ or social aggregate” (Margetts et al. 2015, p. 112).

4 In this paper, we are concerned primarily with institutions conceptualized as something that can be formalized and made explicit, and thus, we do not delve into the “socially extended cognitive institutions” (Ransom and Gallagher 2020) that may appear spontaneously when actors systematically tackle cognitive tasks in a distributed manner.

5 For an example of how an online institution might look like, Daniel Memmi (2014) has discussed Wikipedia as an emerging virtual institution while arguing that subsequent online institutions “need more or less the same ingredients: strong norms, clear rules, a stable social structure, conflict-resolution procedures, and sufficient resources” (2012, p. 81). While we agree in general with the framework proposed by Memmi (2014, and subsequently in 2015), in this paper, we want to draw the reader’s attention to a fundamental difficulty that such online institutions will unavoidably face because of the inherent plurality of online norms.

6 Source: https://blog.twitter.com/en_us/topics/company/2020/2020-election-changes
